# Comparative performance of national civil registration and vital statistics systems: a global assessment

**DOI:** 10.2471/BLT.22.289033

**Published:** 2023-10-19

**Authors:** Lene Mikkelsen, Jessica Hooper, Tim Adair, Azza Badr, Alan D Lopez

**Affiliations:** a LM Consulting, Tamborine Mountain, Australia.; b Manchester, England.; c The Nossal Institute for Global Health, Melbourne School of Population and Global Health, University of Melbourne, Level 2, 32 Lincoln Square North, The University of Melbourne, Victoria, 3010, Australia.; d Division of Data, Analytics and Delivery for Impact, World Health Organization, Geneva, Switzerland.

## Abstract

**Objective:**

To assess the current state of the world’s civil registration and vital statistics systems based on publicly available data and to propose strategic development pathways, including priority interventions, for countries at different levels of civil registration and vital statistics performance.

**Methods:**

We applied a performance assessment framework to publicly available data, using a composite indicator highly correlated with civil registration and vital statistics performance which we then adjusted for data incomparability and missing values.

**Findings:**

Globally, civil registration and vital statistics systems score on average 0.70 (0–1 scale), with substantial variations across countries and regions. Scores ranged from less than 0.50 in emerging systems to nearly 1.00 in the most developed systems. Approximately one fifth of the world’s population live in the 43 countries with low system performance (< 0.477). Irrespective of system development, health sector indicators consistently scored lower than other determinants of civil registration and vital statistics performance.

**Conclusion:**

From our assessment, we provide three main recommendations for how the health sector can contribute to improving civil registration and vital statistics systems: (i) enhanced health sector engagement in birth and death notification; (ii) a more systematic approach to training cause of death diagnostics; and (iii) leadership in the implementation of verbal autopsy methods. Four different civil registration and vital statistics improvement pathways for countries at different levels of system development are proposed, that can constitute a blueprint for regional civil registration and vital statistics strengthening activities that countries can adapt and refine to suit their capabilities, resources, and particular challenges.

## Résumé

**Objective:**

Évaluer l'état actuel des systèmes d'enregistrement des faits d'état civil et de statistiques d'état civil dans le monde en fonction des données accessibles au public et proposer des pistes de développement stratégique, notamment des interventions prioritaires, aux pays présentant différents niveaux de performance en la matière.

**Méthodes:**

Nous avons appliqué un cadre d'évaluation des performances aux données accessibles au public, en utilisant un indicateur composite étroitement lié à l'efficacité des systèmes d'enregistrement des faits d'état civil et de statistiques d'état civil. Nous l'avons ensuite ajusté pour tenir compte de l'incomparabilité des données et des valeurs manquantes.

**Résultats:**

Globalement, le score des systèmes d'enregistrement des faits d'état civil et de statistiques d'état civil s'élèvent en moyenne à 0,70 (échelle 0–1), avec des variations considérables d'un pays et d'une région à l'autre. Les scores vont de moins de 0,50 dans les systèmes émergents à près de 1,00 dans les plus développés. Environ un cinquième de la population mondiale vit dans les 43 pays où le système affiche les plus basses performances (< 0,477). Quel que soit le stade de développement du système, les indicateurs du secteur de la santé ont invariablement obtenu des scores inférieurs à d'autres déterminants des performances d'enregistrement des faits d'état civil et de statistiques d'état civil.

**Conclusion:**

En nous fondant sur notre évaluation, nous formulons trois recommandations majeures pour que le secteur de la santé puisse contribuer à l'amélioration des systèmes d'enregistrement des faits d'état civil et de statistiques d'état civil: (i) une plus grande mobilisation du secteur dans la notification des naissances et des décès; (ii) une approche plus systématique des formations au diagnostic des causes de décès; et enfin, (iii) un meilleur leadership dans la mise en œuvre de méthodes d'autopsie verbale. Nous suggérons également quatre pistes d'amélioration différentes dans l'enregistrement des faits d'état civil et de statistiques d'état civil pour les pays à divers degrés d'évolution en la matière. Elles peuvent servir de modèle aux activités de renforcement de l'enregistrement des faits d'état civil et de statistiques d'état civil, un modèle que les pays peuvent adapter et optimiser en fonction de leurs capacités, ressources et défis spécifiques.

## Resumen

**Objetivo:**

Evaluar el estado actual de los sistemas de registro civil y estadísticas vitales a escala mundial a partir de los datos disponibles públicamente y proponer vías estratégicas de desarrollo, incluidas intervenciones prioritarias, para países con diferentes niveles de rendimiento en materia de registro civil y estadísticas vitales.

**Métodos:**

Se aplicó un marco de evaluación del rendimiento a los datos disponibles al público, mediante un indicador compuesto altamente relacionado con el rendimiento del registro civil y las estadísticas vitales, que luego se ajustó para tener en cuenta la incomparabilidad de los datos y los valores que faltaban.

**Resultados:**

A nivel mundial, los sistemas de registro civil y estadísticas vitales obtienen una puntuación media de 0,70 (escala 0-1), con variaciones sustanciales entre países y regiones. Las puntuaciones oscilan entre menos de 0,50 en los sistemas emergentes y casi 1,00 en los sistemas más desarrollados. Aproximadamente una quinta parte de la población mundial vive en los 43 países con un bajo rendimiento del sistema (<0,477). Independientemente del desarrollo del sistema, los indicadores del sector sanitario obtuvieron sistemáticamente puntuaciones más bajas que otros factores determinantes de los rendimientos del registro civil y las estadísticas vitales.

**Conclusión:**

A partir de la evaluación, se ofrecen tres recomendaciones principales sobre cómo puede contribuir el sector sanitario a la mejora de los sistemas de registro civil y estadísticas vitales: (i) un mayor compromiso del sector sanitario en la notificación de nacimientos y defunciones; (ii) un enfoque más sistemático de la formación en el diagnóstico de la causa de muerte; y (iii) liderazgo en la aplicación de métodos de autopsia verbal. Se proponen cuatro vías diferentes de mejora del registro civil y las estadísticas vitales para países con distintos niveles de desarrollo del sistema, que pueden constituir un anteproyecto de actividades regionales para el fortalecimiento del registro civil y las estadísticas vitales que los países pueden adaptar y perfeccionar según sus capacidades, recursos y desafíos específicos.

## ملخص

**الغرض:**

تقييم الحالة الحالية لأنظمة التسجيل المدني والإحصاءات الحيوية في العالم بناءً على البيانات المتاحة للجمهور، واقتراح مسارات التنمية الاستراتيجية، بما في ذلك التدخلات ذات الأولوية، للدول ذات مستويات مختلفة من أداء التسجيل المدني والإحصاءات الحيوية.

**الطريقة:**

قمنا بتطبيق إطار عمل لتقييم الأداء على البيانات المتاحة للجمهور، وذلك باستخدام مؤشر مركب يرتبط بشكل وثيق بالتسجيل المدني وأداء الإحصاءات الحيوية، والذي قمنا بعد ذلك بضبطه لملاءمة عدم قابلية مقارنة البيانات والقيم المفقودة.

**النتائج:**

على الصعيد العالمي، تسجل أنظمة التسجيل المدني والإحصاءات الحيوية متوسط 0.70 (مقياس من 0 إلى 1)، مع وجود اختلافات ملموسة عبر الدول والمناطق. وتراوحت الدرجات من أقل من 0.50 في الأنظمة الناشئة إلى ما يقرب من 1.00 في أكثر الأنظمة تطوراً. يعيش ما يقرب من خمس سكان العالم في 43 دولة ذات أداء منخفض للنظام (أقل من 0.477). وبغض النظر عن تطور النظام، سجلت مؤشرات القطاع الصحي بشكل متسق درجات أقل من المحددات الأخرى للتسجيل المدني وأداء الإحصاءات الحيوية.

**الاستنتاج:**

وفقًا للتقييم لدينا، نحن نقدم ثلاث توصيات رئيسية حول كيفية مساهمة القطاع الصحي في تحسين أنظمة التسجيل المدني والإحصاءات الحيوية: (1) تعزيز مشاركة القطاع الصحي في الإبلاغ عن الولادات والوفيات؛ و(2) اتباع أسلوب أكثر انتظامًا في التدريب على تشخيص أسباب الوفاة؛ و(3) القيادة في تنفيذ أساليب التشريح السردي. تم اقتراح أربعة مسارات مختلفة لتحسين التسجيل المدني والإحصاءات الحيوية للدول ذات مستويات مختلفة من تطوير النظام، والتي يمكن أن تشكل مخططًا لأنشطة تعزيز التسجيل المدني والإحصاءات الحيوية التي يمكن للدول التكيف معها وتنقيحها لتناسب قدراتها، ومواردها، والتحديات الخاصة بها.

## 摘要

**目的:**

基于公开可用数据评估世界民事登记和人口动态统计系统的现状，并为处于不同民事登记和人口动态统计系统的国家提出战略性发展途径，包括优先干预措施。

**方法:**

我们使用与民事登记和人口动态统计绩效高度相关的综合指标，将绩效评估框架应用于公开的可用数据，然后对没有对比性的数据和缺失值进行调整。

**结果:**

在全球范围内，民事登记和人口动态统计系统的平均得分为 0.70 分（0-1 分），不同国家和地区之间存在很大差异。得分范围从不到 0.50（新开发系统）到接近 1.00（最发达系统）不等。大约五分之一的世界人口生活在 43 个系统性能较低的国家 (< 0.477)。无论系统发展程度如何，卫生部门指标的得分始终低于民事登记和人口动态统计绩效的其他决定因素的分数。

**结论:**

根据我们的评估，我们就卫生部门如何为改进民事登记和人口动态统计系统提出了三项主要建议：(i) 加强卫生部门对出生和死亡通知的参与；(ii) 采用更系统的方法培训死亡诊断；(iii) 领导实施死因推断方法。我们对处于不同系统开发水平的国家提出了四种不同的民事登记和人口动态统计绩效改进途径，这些途径有助于为区域民事登记和人口动态统计制定蓝图，各国可以根据其能力、资源和特殊挑战进行调整和完善以加强相关活动建设。

## Резюме

**Цель:**

Оценить текущее состояние мировых систем регистрации актов гражданского состояния и систем учета естественного движения населения на основе общедоступных данных и предложить стратегические пути развития, включая приоритетные мероприятия, для стран, находящихся на разных уровнях развития системы регистрации актов гражданского состояния и системы учета естественного движения населения.

**Методы:**

Система оценки эффективности была применена к общедоступным данным с использованием комплексного показателя, высоко коррелирующего с показателями регистрации актов гражданского состояния и системы учета естественного движения населения, который затем был скорректирован с учетом несопоставимости данных и недостающих значений.

**Результаты:**

В целом по миру системы регистрации актов гражданского состояния и систем учета естественного движения населения в среднем оцениваются в 0,70 балла (по шкале 0–1), причем в разных странах и регионах эти показатели существенно различаются. Показатели варьировались от менее 0,50 в развивающихся системах до почти 1,00 в наиболее развитых системах. Примерно пятая часть населения Земли проживает в 43 странах с низким уровнем эффективности системы (< 0,477). Независимо от развития системы, показатели сектора здравоохранения неизменно оказывались ниже по сравнению с другими факторами, определяющими эффективность регистрации актов гражданского состояния и системы учета естественного движения населения.

**Вывод:**

По результатам проведенной оценки сформулированы три основные рекомендации по вкладу сектора здравоохранения в совершенствование систем регистрации актов гражданского состояния и систем учета естественного движения населения: (i) расширение участия сектора здравоохранения в процессе оповещения о случаях рождения и смерти; (ii) более системный подход к обучению диагностике причин смерти; (iii) лидерство во внедрении методов вербальной аутопсии. Предлагаются четыре различных пути совершенствования регистрации актов гражданского состояния и системы учета естественного движения населения для стран, находящихся на разных уровнях развития системы, которые могут стать основой для региональных мероприятий по укреплению системы регистрации актов гражданского состояния и системы учета естественного движения населения, при этом страны могут адаптировать и совершенствовать их с учетом своих возможностей, ресурсов и конкретных задач.

## Introduction

Fundamental to the assessment of population health is accurate and timely information on patterns and trends in human fertility and mortality.^1^ Almost all countries have a method to record vital events; however, not all have systems which can reliably determine cause of death and many have systems that fail to record all, or even most, births and deaths that occur. In countries with well-developed civil registration and vital statistics systems, the data they provide on vital events are crucial in assisting national policy-makers and administrators with decision-making, in sectoral strategy formulation, and public health policy and planning.^2^ Given the multisectoral demand for such data, the successful stewardship of countries is thus highly dependent on a functioning national civil registration and vital statistics system which provides reliable and timely vital statistics.

The health sector has a particularly strong need for functional civil registration and vital statistics systems. The recent coronavirus disease 2019 (COVID-19) pandemic has shown us that we need accurate and timely records of deaths and cause of death to assess, for example, the effectiveness of prevention and control strategies. The management and evaluation of disease reduction also requires reliable and timely data on age- and cause-specific mortality from well-functioning civil registration and vital statistics systems. The universal commitment of governments in 2015 to the United Nations (UN) sustainable development goals, which depends, in part, on data from vital statistics systems for monitoring progress, has helped highlight the need for reliable, continuous and comparable vital statistics, creating momentum to improve the recording of fertility and mortality data.

The need to know the number of children born every year, as well as the number of people dying and what they died from, has stimulated several global, regional and national responses over the past two decades to strengthen civil registration and vital statistics systems.^3^^,^^4^ Numerous tools and frameworks were developed, and applied, to assist countries to identify various challenges with their civil registration and vital statistics systems and decide on improvement strategies and interventions.^5^^–^^8^ The primary limitation of these earlier efforts is their lack of comparability arising from their dependence on self-assessment to generate data and information. We present an alternative statistical framework that relies solely on available data to classify countries into categories of civil registration and vital statistics system performance. Based on these classifications, we propose for each category a strategic development pathway with some priority interventions and improvement actions they can undertake to improve their systems’ development. 

## Methods

To assess system performance, we used a performance assessment framework developed through consultation with civil registration and vital statistics experts and stakeholders. This framework identifies, as parsimoniously as possible, the key elements of a civil registration and vital statistics system which defines best practices for well performing systems. The framework comprises four domains: (i) enabling environment for civil registration and vital statistics; (ii) functioning of the civil registration and vital statistics system; (iii) health sector contributions to civil registration and vital statistics; and (iv) dissemination practices and demand for vital statistics (Table 1). Performance within each domain is measured using a series of indicators listed in Table 1. Three databased outcome indicators were used to reflect system functionality: (i) birth registration completeness; (ii) death registration completeness; and (iii) percentage of deaths with a usable cause available from the companion articles.^9^^,^^10^ We calculated birth registration completeness as registered births divided by the UN World Population Prospects’ estimate of live births.^9^^,^^11^ In some countries, the denominator used was the estimates from the Global Burden of Disease database produced by the Institute of Health Metrics and Evaluation.^12^ Death registration completeness is defined as the extent to which the civil registration and vital statistics system manages to count all deaths that occur in a year; and in many countries it is calculated using the Adair-Lopez empirical completeness method, which estimates completeness of death registration using a statistical model and several covariates.^10^^,^^13^ In countries where the empirical completeness method is less accurate because of high death rates due to acute immunodeficiency syndrome or violence, conflict and war, estimated deaths are used as the denominator.^11^ We calculated the percentage of deaths with a usable cause as the percentage of estimated total deaths that have a cause as per the International Medical Certificate of Cause of Death, and for which the assigned cause is not classified as a nonspecific or garbage code.^14^

**Table 1:** Domains and indicators used for the civil registration and vital statistics performance assessment framework

Domain	Description	Indicator
Domain 1: enabling environment for civil registration and vital statistics	Assess strength of fundamental support mechanisms required for an effective functioning of a civil registration and vital statistics system	1.1 Status of legislation on birth and death registration 1.1a Policies to ensure free birth registration 1.1b Policies to ensure free death registration
		1.2 Status of legislation on medical certification of death
		1.3 Civil registration and vital statistics assessment activity
		1.4 Status of national civil registration and vital statistics coordination mechanism
		1.5 Status of national civil registration and vital statistics improvement plan or strategy
Domain 2: functioning of the civil registration and vital statistics system	Assess the operation and efficiency of a well-functioning system	2.1 Transmission of birth and death records from regional offices to central storage facility
		2.2 Access to civil registration offices or registration points
		2.3 Training of registrars
Domain 3: health sector contributions to civil registration and vital statistics	Measure the extent of application of international health standards and practices to ascertain the involvement of the health sector in civil registration and vital statistics functions	3.1 Use of standard WHO International medical certificate of cause of death for reporting
		3.2 Use of ICD-compliant medical certification of cause of death
		3.3 Training in medical certification
		3.4 Data quality checks for cause of death data
		3.5 Qualifications of staff in ICD coding
		3.6 Verbal autopsy implementation status
		3.7 Cause of death statistics
Domain 4: dissemination practices and demand for vital statistics	Evaluate whether vital statistics are routinely compiled and disseminated. Their quality and timeliness	4.1 Vital statistics report publication frequency
		4.2 Availability of annual numbers of births disaggregated by sex, age, and geographic or administrative region
		4.3 Availability of annual numbers of deaths disaggregated by sex, age, and geographic or administrative region
		4.4 Availability of annual vital statistics at the national and subnational levels
		4.5 Consistency and plausibility checks on fertility and mortality
		4.6 Delay between reference year and the time when detailed national statistics on causes of death by sex and age are made available to the public
		4.7 Use of vital statistics for policy and programme purposes

ICD: *International statistical classification of diseases and related health problems*; WHO: World Health Organization.

### Data sources

The information used in our assessment of civil registration and vital statistics systems is drawn from a desk review of publicly available sources from international agencies and databases, country websites and published literature.^6^^,^^8^^,^^15^^–^^18^ Key data sources are: World Health Organization (WHO) SCORE country assessment summaries; WHO rapid/comprehensive civil registration and vital statistics assessments; UN Economic Commission for Asia and the Pacific mid-term questionnaires; United Nations Children’s Fund civil registration and vital statistics country profiles; World Bank Identification for Development data; and regional and country civil registration and vital statistics status and/or progress reports.^9^^,^^10^

### Data analysis

Using a variety of data sources for the indicators of the performance assessment framework naturally poses a challenge when calculating civil registration and vital statistics system performance; for example, different sources might not use the same response categories or respondents, and/or time periods may vary affecting the comparability of scores. Based on the data available for each country we first calculated an overall assessment score for each indicator, equally weighting each source. Next, given the issues of data comparability and subjectivity identified above, we adjusted the overall assessment score using an external summary score, based on outcome indicators and other variables likely to affect system performance. The resulting adjusted overall assessment scores were produced for each indicator, domain, and across all domains of the performance assessment framework for all Member States.

For each domain in the performance assessment framework, we estimated a predicted adjusted overall assessment score based on a statistical model of the functional relationship between the external summary score and the adjusted overall assessment score for countries with available data. Details of this regression model and its predictive validity are available in our online repository.^19^

Based on the adjusted overall assessment scores across all domains of the performance assessment framework, all 194 WHO Member States were classified into four performance levels (Box 1).

Box 1 **Definition of different performance levels of civil registration and vital statistics systems**

- High performance: adjusted overall assessment score over 0.871
- Moderate performance: adjusted overall assessment score from 0.677 up to 0.871
- Weak performance: adjusted overall assessment score from 0.484 up to 0.677
- Low performance: adjusted overall assessment score less than 0.484

We chose the threshold values of each performance category as follows: (i) high performance systems are those where the adjusted overall assessment score was within 90% of the maximum observed (excluding predicted) adjusted overall assessment score attained by a country with more than one million inhabitants; (ii) moderate performance is at least 70% but less than 90% of the maximum adjusted overall assessment score; (iii) weak performance is at least 50% but less than 70% of the maximum adjusted overall assessment score; and (iv) low performance is less than 50% of the maximum adjusted overall assessment score. We used this classification scheme to assess both overall performance and individual domains. 

## Results

**Fig. 1:**
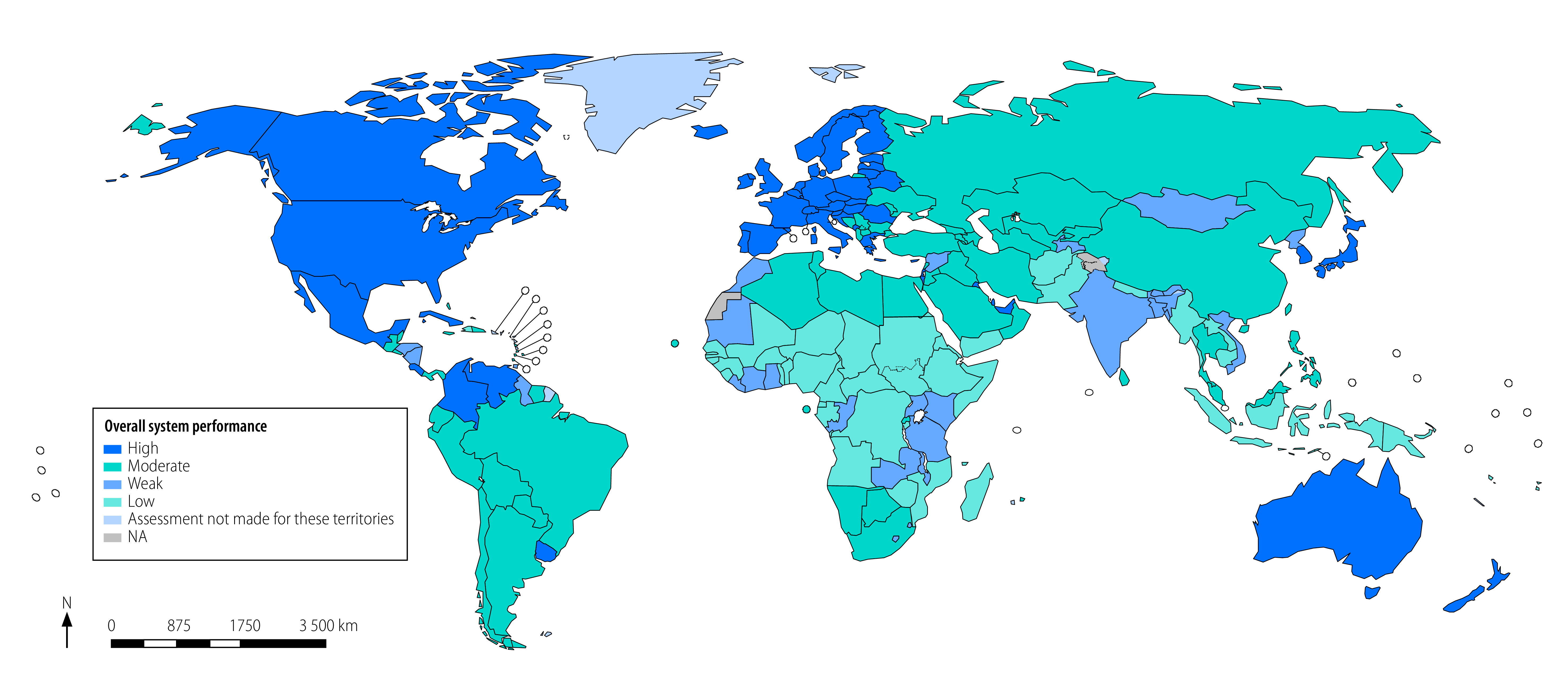
Average adjusted overall assessment score of civil registration and vital statistics systems for all WHO Member States, 2019

NA: not applicable; WHO: World Health Organization.

Note: Overall performance is an average of the domains: (i) enabling environment for system; (ii) functioning of the system; (iii) health sector contributions to civil registration and vital statistics; (iv) dissemination practices and demand for vital statistics. Countries with a population higher than 1 million were included in the calculation of the maximum score achieved. High performance is designated to countries with an adjusted overall assessment score that is 90% or more of the maximum score; moderate performance is designated to countries with an adjusted overall assessment score that is at least 70% but less than 90% of the maximum score achieved; weak performance is designated to countries with an adjusted overall assessment score that is at least 50% but less than 70% of the maximum score achieved; and low performance is designated to countries with an adjusted overall assessment score that is less than 50% of the maximum score achieved.

The assessment showed that 121 countries (an estimated 55% of the world’s population) have civil registration and vital statistics systems of high or moderate performance (Table 2). The system performance was assessed as weak in 30 countries where approximately one quarter of the world’s population live. As such, one fifth of people live in 43 countries with the poorest-performing systems. Globally, the average adjusted overall assessment score was 0.699; ranging from 0.361 on average in countries classified as low, to 0.926 on average in countries rated as high performance. More detail on the adjusted overall assessment score for individual countries can be found in the online repository. Fig. 1 shows the classification of countries based on their respective average adjusted overall assessment scores across all domains.

**Table 2:** Stratification of WHO Member States and population according to civil registration and vital statistics system performance categories, 2019

Overall performance level	No. of WHO Member States	% of global population^11^	Average adjusted overall assessment score and range
High	53	18	0.926 (0.871–0.984)
Moderate	68	37	0.787 (0.696–0.867)
Weak	30	26	0.581 (0.495–0.667)
Low	43	19	0.361 (0.093–0.477)
**All levels**	**194**	**100**	**0.699 (0.093–0.984)**

WHO: World Health Organization.

Of the 53 systems with high performance, most were located in the Region of the Americas and European Region, with very few in the Western Pacific or Eastern Mediterranean Regions. Countries in the high category have systems which record all, or almost all, vital events and where it is compulsory for medical doctors to certify causes of death before a death can be registered, and to use rules and standards from the International Statistical Classification of Diseases and Related Health Problems (ICD).

The 68 countries with moderately performing systems manage to record most, or often all, births and deaths but efficiency and data quality are of concern. In many cases, listed causes of death are either incorrectly certified or coded. As a result, confidence in the data is reduced among data users and stakeholders.

Countries classified as having weak system performance register the majority of events that occur in urban areas, hospitals and health facilities. However, outside of cities, deaths, and in particular child deaths, are grossly underreported. Cause of death is often not medically certified nor determined using standard verbal autopsy methods. These challenges are common in the Africa and South-East Asia Regions and among the smaller countries situated in the Region of the Americas and the Western Pacific Region.

Most of the 43 countries classified as having low-performing systems have only recently begun to develop their civil registration and vital statistics systems, and usually only have registration points in urban areas. The data produced by such systems is generally incomplete and does not include cause of death. 

More detailed information about the performance of the four categories of countries can be obtained from their scores on each of the four domains of the performance assessment framework (Table 3). Globally, the weakest aspect of all civil registration and vital statistics systems is domain 3 (health sector contributions to civil registration and vital statistics), with an average adjusted overall assessment score of 0.651, and for 50 countries it was classified as a low adjusted overall assessment score (0.268; Table 3). The country data on the average score for each domain is available in the online repository.^19^

**Table 3:** Number of WHO Member States in each performance category and average adjusted overall assessment score of civil registration and vital statistics systems in each domain, 2019

Domain	No. of WHO Member States (average adjusted overall assessment score per domain)				
	Performance				All Member States
	High	Moderate	Weak	Low	
Domain 1: Enabling environment for civil registration and vital statistics	53 (0.940)	68 (0.793)	49 (0.602)	24 (0.355)	194 (0.731)
Domain 2: Functioning of the civil registration and vital statistics system	55 (0.946)	69 (0.811)	19 (0.607)	51 (0.295)	194 (0.694)
Domain 3: Health sector contributions to civil registration and vital statistics	50 (0.926)	60 (0.773)	34 (0.596)	50 (0.268)	194 (0.651)
Domain 4: Dissemination practices and demand for vital statistics	50 (0.936)	74 (0.811)	38 (0.596)	32 (0.315)	194 (0.719)

WHO: World Health Organization.

## Discussion

Our results indicate that for 38% of WHO Member States, civil registration and vital statistics system performance is well below the level required for reliably informing policy and programme management. Our results identify a major deficit in domain three (that is, contributions of the health sector to strengthening civil registration and vital statistics systems), particularly in countries with weak- and low-performing systems. Health facilities and community health workers in these countries are frequent points of contact with the population, and could be given greater responsibility for notifying vital events to registration authorities. Similarly, since the diagnostic accuracy and quality of mortality data should be a key concern for the health sector, a more active role of physicians in health institutions in ensuring that the certification practices are of sufficient quality to meet policy needs would be desirable.

The experiences of Member States with civil registration and vital statistics system building and strengthening over the past two decades provide compelling evidence that change is possible but requires long-term commitment from governments to be realized.^20^^–^^22^ There is a substantial body of knowledge that has been accumulated by experts over the years that can be used to improve civil registration and vital statistics practice; we propose a generic progress pathway for each of the four civil registration and vital statistics performance categories that can be adapted to individual country contexts, with key priority actions appropriate for different system performance levels. These are summarized in Table 4.

**Table 4:** Civil registration and vital statistics improvement pathways for countries of different system performance levels

Areas for improvement and suggested actions	Performance level			
	Low	Weak	Moderate	High
**Governance and stewardship**				
Advocate with national and local authorities for improved civil registration and vital statistics	Yes	Yes	Yes	No
Establish a coordination mechanism for civil registration and vital statistics improvement	Yes	Yes	No	No
Review resource needs for improving civil registration and vital statistics systems	Yes	Yes	Yes	No
Review legal and regulatory frameworks for civil registration and vital statistics	Yes	Yes	No	No
Review functioning of existing coordination mechanisms	No	Yes	Yes	No
Conduct business process mapping for all vital events	Yes	Yes	No	No
Develop publicity and incentive schemes for vital event registration	Yes	Yes	No	No
Conduct targeted information and education campaigns	Yes	Yes	No	No
Set up birth and death registration points in larger hospitals	Yes	Yes	No	No
Conduct a comprehensive assessment of all aspects of current system selected for improvement, develop a prioritized and costed improvement plan for these	No	Yes	Yes	Yes
Develop a monitoring plan to assess the civil registration and vital statistics system progress	No	Yes	Yes	Yes
Determine for which areas technical assistance is needed	Yes	Yes	Yes	No
**Vital events notification, transmission of data and digitalization**				
Develop or update manuals for civil registrars	Yes	Yes	Yes	No
Develop or update manuals for the notification of vital events by the health sector	Yes	Yes	Yes	No
Review vital event notification and registration forms for computerization	Yes	Yes	Yes	No
Translate registration forms into local languages	Yes	Yes	No	No
Institute or review birth and death notification procedures in health facilities	Yes	Yes	Yes	Yes
Generate procedures for active notification of community deaths	Yes	Yes	No	No
Progressively introduce computerization for all civil registration and vital statistics procedures	Yes	Yes	Yes	No
Increase coverage of under-served areas and groups	No	Yes	Yes	No
Investigate the use of mobile phones for notifying vital events at community level	Yes	Yes	No	No
**Strengthening cause of death data practices in health facilities and the community**				
Conduct reviews into quality of death certification	No	No	Yes	Yes
Develop training materials for physicians to correctly certify causes of deaths	Yes	Yes	Yes	Yes
Conduct reviews of cause of death coding quality	No	No	Yes	Yes
Strengthen skills of staff for ICD mortality coding	Yes	Yes	Yes	Yes
Introduce centralized ICD mortality coding	Yes	Yes	No	Yes
Introduce, where needed, automated verbal autopsy for community deaths	Yes	Yes	No	No
Introduce sample registration with verbal autopsy in representative areas	Yes	Yes	No	No
Introduce automated software for transferring vital statistics data from health facilities to central storage facilities	Yes	Yes	Yes	Yes
**Data compilation, analysis and dissemination**				
Compile, assess and analyse available data to produce statistics	Yes	Yes	Yes	Yes
Use data from health and demographic surveillance sites to estimate levels of fertility and mortality	Yes	Yes	No	No
Introduce automated ICD coding software where possible	No	No	Yes	Yes
Strengthen skills of staff to analyse vital statistics data and verify their quality	Yes	Yes	Yes	Yes
Publish annual vital statistics reports	No	Yes	Yes	Yes
Assess the quality of cause of death data using an automated software	No	Yes	Yes	Yes
Facilitate online access to vital statistics data	No	Yes	Yes	Yes
Use global standards for the compilation of vital statistics	Yes	Yes	No	No
Improve timeliness and dissemination of vital statistics data	No	Yes	Yes	Yes
Introduce regular quality control mechanisms for vital statistics data	Yes	Yes	Yes	Yes

ICD: *International statistical classification of diseases and related health problems*.

According to our performance typology, the countries with high-performing systems already have civil registration and vital statistics systems that capture all, or almost all, births and deaths, and ensure that the WHO standard international death certificate is used for certifying the cause. Yet improvements in diagnostic accuracy and specificity are still possible for many of these countries through better training of doctors in correct death certification. Other potential gains could be achieved through routine application of data quality software, for example, ANACONDA (co-developed by the University of Melbourne, Melbourne, Australia and the Swiss Tropical and Public Health Institute, University of Basel, Basel, Switzerland) to check the accuracy and completeness of mortality and cause of death data.^23^ Finally, introduction of electronic notification and registration will lead to improved system efficiency in countries where this is not yet available (Table 4).

In the countries where system performance was classified as moderate, most vital events are registered, but quality and timeliness of the cause of death data are often poor due to substandard certification practices among doctors.^24^ There is often limited awareness by government and health authorities of data deficiencies and hence little interest in conducting data quality reviews. Introduction of electronic data deficiency analysis and introduction of automated ICD coding would dramatically improve the quality of cause of death data and speed up data reporting and dissemination.^25^ The pathway suggested in Table 4 for this category therefore includes system improvements aimed at more accurate and timely data collection for policy. 

Most countries in the weak- and low-performance categories have conducted assessments of their civil registration and vital statistics systems, but frequently only managed to implement a few steps in their improvement plans; often lacking financial, technical and human resources. The differences between their improvement strategies are detailed in Table 4 and, while many actions are similar, their scope will differ according to development level. Experience suggests introducing improvement actions in a stepwise fashion is crucial; initially in a pilot population or area before attempting national implementation. A key priority for countries initiating system development is intersectoral coordination, which can guide implementation and find agreement on specific interventions and development timeframes.

A functioning and efficient system requires a full understanding of all the steps involved in registering vital events, hence the importance of conducting a detailed business process mapping to ensure that all potential synergies in the system are explored, duplications identified and resolved, and problems mitigated. Priority should be given to improving the percentage of all vital events which are notified and registered each year. A starting point could be a review of the legal and regulatory frameworks for civil registration and vital statistics; including what types of incentives or penalties would be appropriate in each given setting. The most effective and sustainable schemes for improving vital event registration are those that link government services to possession of registration papers, such as a birth certificate for obtaining identification cards; establishing citizenship; proof of age; family relationships; election enrolment and passports; and death certificate for claiming benefits, insurance and inheritance. The application of a formal performance assessment framework demonstrates that it is possible to measure current civil registration and vital statistics development levels both globally and for individual countries. Together, governments and development partners can inspect the scores for individual domains and decide on which actions are necessary and suitable for local conditions.

While universal medical certification is not feasible in countries with poorly performing or burgeoning systems, countries should be encouraged to implement validated, cost-efficient verbal autopsy methods that can generate evidence on the leading causes of community deaths. Similarly, the health sector could obligate their facilities to report inpatient births, deaths and causes of deaths on standard forms to registration authorities. If collaborating with local registration authorities, this approach should be relatively easy to implement. 

The COVID-19 pandemic disrupted functioning civil registration and vital statistics systems, preventing many countries from reliably estimating deaths, especially in countries where the civil registration and vital statistics system is paper-based and/or dependent on in-person visits.^26^ One survey revealed that just 24% of countries (16 of 66 countries) had uninterrupted civil registration and vital statistics during the pandemic; while just 73 of 194 (38%) countries had national data complete enough to accurately measure excess mortality.^26^^,^^27^ One positive trend to emerge from the pandemic is the increased use of online registration services to register births and deaths. The COVID-19 pandemic further highlights the importance of strong and efficient civil registration and vital statistics systems that can correctly count the deaths and measure the true impact of future epidemics.^28^^–^^30^

The primary limitation of our study is the wide range of disparate data sources used to measure civil registration and vital statistics system performance. As they all had some information content, we gave equal weight to each data source. Although we assessed all data accessible to our team, it is likely that more information was available locally that could have had an impact on the adjusted overall assessment scores. As such, the results presented here are likely more pessimistic than reality. Second, data collected for other purposes might not translate perfectly to the indicators contained in our performance assessment framework. As such, they might sometimes only provide an approximate measurement of the domain characteristic under investigation. Furthermore, the date and period of data collection varied by almost a decade (2010 to 2020) across data sources, potentially increasing bias towards more favourable responses from recent data collection efforts. These limitations need to be considered when monitoring the implementation and evaluating the effectiveness of strategies suggested by our framework.

In conclusion, governments need to empower the health sector to become active participants in civil registration and vital statistics systems, especially as they pertain to the notification of births and deaths and cause of death determination. The health community can contribute to the improvement of medical certification practices and coding expertise with automated ICD coding tools. In countries where the majority of individuals die at home without medical supervision, the health sector needs to provide leadership for the implementation of cost-effective verbal autopsy methods and other strategies to increase knowledge about leading causes of home death^31^.

The tools, methods and accumulated knowledge and expertise now exist to implement the interventions proposed in the four civil registration and vital statistics improvement pathways. The WHO *Civil registration and vital statistics strategic implementation plan, 2021–2025*^32^ can provide the organizational framework and health sector leadership to facilitate appropriate, best-practice technical assistance to countries, especially those with weak- or low-performing systems, to rapidly and sustainably improve performance. While financial resources are vital to this process, real progress will only be made if there is strong leadership and commitment from countries to improve their registration systems in recognition of the health benefits for their populations.
